# The validity of the residuals approach to measuring resilience to adverse childhood experiences

**DOI:** 10.1186/s13034-022-00449-y

**Published:** 2022-03-01

**Authors:** Stephanie Cahill, Reinmar Hager, Tarani Chandola

**Affiliations:** 1 Evolution, Infection and Genomics, Faculty of Biology, Medicine and Health, Manchester Academic Health Science Centre, University of Manchester, Manchester, UK; 2 Faculty of Humanities, Cathie Marsh Institute for Social Research, University of Manchester, Manchester, UK; 3 Methods Hub, Department of Sociology, Faculty of Social Sciences, University of Hong Kong, Hong Kong, People’s Republic of China

**Keywords:** Resilience, Residuals, Measurement approaches, Adverse childhood experiences, ALSPAC

## Abstract

**Background:**

Resilience is broadly defined as the ability to maintain or regain functioning in the face of adversity. Recent work to harmonise the quantification and definition of resilience quantifies resilience as the residual variance in psychosocial functioning that remains after accounting for adversity exposure. However, there have been no published studies that have formally investigated the validity of this approach. Considering this, we examine the construct and predictive validity of the residuals approach using participants from the Avon Longitudinal Study of Parents and Children (ALSPAC), a multigenerational, longitudinal cohort study.

**Methods:**

We regressed exposures of adolescent adversity on adolescent psychopathology scores using the Strength and Difficulties Questionnaire and obtained the residual variance. We investigated construct validity by analysing whether previously identified demographic and resilience factors significantly predicted resilience. Predictive validity of resilience was investigated by comparing the predictive power of resilience with other determinants of psychosocial functioning on two developmental outcomes: depressive symptoms at 18 years, measured by the Short Moods and Feelings Questionnaire, and NEET (Not in Employment, Education or Training) status at 17 and 23 years. The associations between depressive symptoms at 18, resilience, ACEs and covariates were tested using multiple linear regression. NEET status at 17 and 23 were run as separate binary multiple logistic regression models to test associations with resilience and known demographics previously associated with NEET status.

**Results:**

Seven previously identified protective factors, including self-esteem, positive sibling relationship, temperament, and positive perception of school, significantly predicted resilience to adolescent psychopathology, thus providing strong construct validity. Resilience significantly predicted a reduction in depressive symptoms at 18 years, and significantly decreased the likelihood of having NEET status at both 17 years and 23 years, even after taking into account early childhood adversity and other risk factors. None of the socioeconomic factors were significantly associated with resilience.

**Conclusions:**

Our study demonstrates that the residuals method of operationalising resilience has good construct and predictive validity yet recommend replication studies. It has the potential to advance research into the mechanisms and modifiability of resilience.

*Trial Registration:* Not applicable.

**Supplementary Information:**

The online version contains supplementary material available at 10.1186/s13034-022-00449-y.

## Background

Some individuals do not develop stress-related disorders despite exposure to childhood adversity. This ‘resilience’ is a well-recognised phenomenon, yet there is considerable variation in the way resilience is defined, operationalised and measured in the literature. An early perspective in resilience research identified resilience as a characteristic or trait of the individual (e.g. [90, 92]) and considered resilient children to be exceptional individuals [5]. However, this static definition of resilience limits the possibility that resilience can vary across situations and develop through the life-course. An emerging consensus in the field is that resilience refers to the dynamic process of adaptation to adversity across the life span, dependent upon context and resources, which enables the individual to successfully negotiate adversity [16, 53, 85]. The process of resilience is thus central to normal development resulting from an individual’s interactions with a range of environmental factors [9, 68]. This definition also assumes that resilience can only be measured after exposure to adversity, as opposed to viewing resilience as a purely intrinsic trait.

Given the negative impact of adverse childhood experiences (ACEs) on a broad range of different physical and mental health aspects [31, 47], resilient functioning after exposure to ACEs should be inferred and measured from functioning across different social, emotional, cognitive and/or behavioural domains. By considering the range in severity of ACEs, resilient functioning refers to better psychosocial functioning compared to others with a similar degree of exposure to ACEs. For example, two individuals could have a similar level of moderate functioning, but the individual with a severe history of exposure to ACEs would have a higher level of resilience, when compared to an individual with moderate or low level exposure to ACEs [49].

### Residuals approach to measuring resilience

The “residuals” approach to measuring resilience is an emerging framework that quantifies resilient functioning as doing better than expected given the degree of ACE exposure. Here, the residual scores of the regression models reflect individual degrees of resilient functioning across different domains, taking into account ACE exposure. Thus, resilience is conceptualised as the extent to which an individual is functioning better than expected given their level of adversity exposure (see [2, 10, 17, 20, 25, 72, 86, 97] for a similar approach, with extensive discussion of this method in [49]).

The conceptual basis for the residuals approach rests on the assumption that psychosocial functioning is determined by multiple factors, and that the independent contributions of these determinants can be estimated. One key determinant is exposure to ACEs. Measurements of exposure to adversity are sensitive to the timing, chronicity and severity of ACEs. Yet, substantial variance in psychosocial functioning outcomes remains often unexplained by ACEs. Resilient functioning after adversity is facilitated by protective resilience factors (RFs) that support individuals to adapt and recover from ACEs [33]. The wider literature suggests that RFs at the individual, family and community level are associated with a reduced likelihood of developing psychosocial problems [23, 33, 87].

Our residuals approach to measuring resilience in this study involves an empirical decomposition of variance in an outcome variable (ratings of child psychopathology from the Strength and Difficulties questionnaire (SDQ) [37], in this case), into components explained by measures of adolescent ACE exposure and a component that is independent of these ACE exposures. The latter residual component captures individual differences in resilience that are not explained by measured variables and provides our putative measure of resilience. That is, individuals with high scores on this component perform better than expected and those with low scores perform worse than expected, corresponding to previously defined resilience and vulnerability. It is a measure of the current resilience of an individual, where resilient functioning refers to better mental wellbeing compared to other individuals with similar ACE exposures.

This approach, in a sense, defines resilience as the sum of unmeasured sources of variance in psychosocial functioning, which is not explained by ACE exposure. A limitation of this approach is that the derived measure of resilience will be influenced by the specific variables used and, consequently, different measures of resilience are possible for a specific person at a specific time. However, we are not primarily interested in deriving a singular measure of resilience, but rather in establishing whether the residuals approach provides a useful measure for operationalising resilience. In order to address this, we need to gather evidence to support the use and interpretation of this measure of resilience.

The residuals approach provides a relatively simple way of measuring resilience in relation to ACEs and psychosocial functioning, quantifying resilience on a continuum of resilience to vulnerability. However, the validity of this methodology has not yet been examined.

### Construct and predictive validity

Resilience is an unobservable, hypothetical construct. The challenge lies in how to measure resilience in a robust way that enables significant advances in the field. Cronbach and Meehl’s [21] seminal article, “Construct Validity in Psychological Tests”, argued that there must be evidence that a measure of a given construct relates to measures of other constructs in a theoretically predictable way [21]. Validity analyses are common approaches to assessing psychometric quality [103] and quantifying a measure’s predictive ability for a key outcome or association with a theoretically relevant construct can provide further support. Predictive validity of resilience must show predictive correlations with later positive psychosocial outcomes.

In our study, we first investigated the construct validity of resilience, when measured using the residuals approach, by analysing whether previously identified RFs and demographic factors significantly predict resilience as measured by the residuals approach. Specifically, we hypothesize that previously theorised resilience factors and associated demographic variables in childhood significantly predict resilience to SDQ total difficulties at 16 years of age.

Second, predictive validity was assessed by comparing the predictive power of the residuals measure of resilience with other determinants of psychosocial functioning on two developmental outcomes. The first, proximal, outcome is a measure of depressive symptoms at age 18, as measured by the Short Mood and Feelings Questionnaire (SMFQ) [3]. The SMFQ is designed to examine the presence of depressive symptoms via a 13-item self-reported questionnaire and is well validated for epidemiological studies [93]. We predict that resilience to SDQ difficulties at 16 years significantly decreases the risk of depressive symptoms at 18 years.

The second, distal, outcome is NEET (Not in Employment, Education or Training) status at 17 and 23 years. NEET status demonstrates an objective and ecologically valid measure of poor functioning (Organisation for Economic Co-operation and [75]. Here, we predict that resilience to SDQ difficulties at 16 years significantly reduces the risk of having NEET status at 17 years and 23 years.

## Method

### Participants

The sample comprised 15,454 participants from the Avon Longitudinal Study of Parents and Children (ALSPAC), a multigenerational, longitudinal cohort study that recruited pregnant women resident in the former Avon Health Authority in south-west England who had an estimated due date between 1st April 1991 and 31st December 1992 [11, 32]. After data cleaning, our sample comprised of 14,694 participants. The study website with detailed information about ALSPAC is available at (http://www.bristol.ac.uk/alspac/), which includes a fully searchable data dictionary and variable search tool (http://www.bristol.ac.uk/alspac/researchers/access/).

### Exposure to adverse childhood experiences (ACEs)

Mothers, partners, and the study child were asked 87 questions about the child’s exposure to 15 ACEs between the ages of 11 and 16 years (Table 1). These adversities were all prospectively measured from questionnaires with the cohort child’s mother, the mother’s partner or the cohort child themselves, or collected at four clinic sessions the children attended between 11 and 15.5 years (see Table 1 for further information on the definitions of each ACE with the timing of collection, type of data collection and number of contributing questions). While there is currently much debate within ACE research about what constitutes ‘adversity’ and the controversial application of ACE scores used at the individual level to predict ill health (see [60, 70], within the present study ACE constructs were derived as binary measures of exposure as described by Houtepen and colleagues for specific use within the ALSPAC cohort, to encourage replication [45].

**Table 1:** Description of variables and data sources used to derive binary measures of adolescent ACEs

ACE	Description	Data Source
Bullying	Five variables on frequency someone threatened/blackmailed, told lies about, put down, upset, peer pressured teenager	Teen Focus Research clinic at 12.5years and 15.5years. Child completed questionnaire 16years
Emotional abuse	Four variables asking if parent or partner has been emotionally cruel to the study child	Mother and partner completed questionnaires at 11years 2months
Emotional neglect	17 variables asking a range of questions on interactions with parent/carers	Teen Focus Research clinic at 12.5years, 13.5years and 15.5years. Child based and child completed questionnaires 16years
Financial difficulties	Six variables on homelessness and inability to pay for food/heating	Mother and partner completed questionnaires at 11years 2months
Parent mental health problems or suicide attempt	14 variables on diagnosed mental health problems, suicide attempts, self harm, and medication for anxiety or depression	Mother, partner and child completed questionnaires at 11years 2months, 12years 1month and 16years
Neighbourhood satisfaction	Four variables of neighbourhood satisfaction	Teen Focus research clinic at 15.5years and child completed questionnaire at 14years
Parent child bond	Three variables measuring how close the partner or mother feels to the study child and a clinic-based measurement of adult/child harmonious interactions	Teen Focus research clinic at 12.5years. Mother and partner completed questionnaires at 12years 1month
Parent convicted offence	Four variables on whether parent or carer convicted of offence	Mother and partner completed questionnaires at 11years 2months and 12years 1month
Parental separation	Six variables on whether parents had divorced or separated	Mother, partner, and child completed questionnaires at 11years 2months and 16years
Physical abuse	Four variables asking if parent or partner has been physically cruel to the study child	Mother and partner completed questionnaires at 11years 2months
Social support—child	Eight variables on number of close friends and satisfaction with friendships	Teen Focus Research clinic at 12.5years, 13.5years and 15.5years
Social support—parent	Two variables on whether the parent has anybody to share feelings with	Mother and partner completed questionnaires at 12years 1month
Household substance abuse	One variable asking if respondent has ever had a drug addiction	Partner completed questionnaire at 11years 2months
Violence between child and partner	Six variables on violence in a romantic relationship	Teen Focus research clinic at 13.5years
Violence between parents	Two variables on physical cruelty between parents	Mother and partner completed questionnaires at 11years 2months

### Strength and difficulties questionnaire

The SDQ is one of the most commonly used ratings of child psychopathology in epidemiological studies [37]. The SDQ questionnaire was maternal-reported at 16 years 6 months and comprises 20 items relating to four different psychosocial scales: emotional symptoms; conduct problems; hyperactivity/inattention and peer problems. Responses are scored using a three-point Likert scale and the answer summed to give a total difficulties score out of 40.

### Short mood and feelings questionnaire

The SMFQ was self-reported by the study child at 18 years 6 months. The SMFQ is designed to examine the presence of depressive symptoms via a 13-item self-reported questionnaire and is well validated [93]. As each question is scored between 0 and 2, the resulting summary score can range between 0–26 with higher scores being more indicative of higher depression. The SMFQ has shown good validity and reliability for scores of 11 or more determining the presence of depression [4].

### Resilience

In order to investigate the validity of the residuals approach, we regressed binary exposures of ACEs experienced in adolescence (11–16years) on the total difficulties score of the SDQ at 16 years 6 months. We extracted the residuals scores as these reflect a spectrum ranging from risk to resilient functioning, i.e., the extent to which an individual has better, or worse, SDQ outcomes than the average score expected given their exposure to ACEs over adolescence.

### NEET status at 17 and 23

NEET status was assessed using the same self-report questionnaire at 17 and 23 years, which included two questions on whether the participants were currently enrolled in any education/training programme or employed. A binary variable was created, which classified the young person as NEET if the answer to both questions was negative, in line with the definition used by the Office for National Statistics [74].

### Resilience factors

Potential individual, family and community factors associated with resilience were chosen based on previous literature [52, 58]. At the individual level these include higher IQ [39, 76], an easy temperament [67], internal locus of control [8], high mental flexibility [81], high self-esteem [76], high linguistic ability [100] and high cognitive skills [7, 42]. At the family level these include attachment to grandparent [99], maternal parenting [42, 88] and sibling relationship [58]. At the community level these include high school engagement [43, 102], positive perception of school [79], engagement with religion [43, 79], regular engagement in extracurricular activity [77] and supportive friendships [38, 40]. See Additional file 1: Table S1 for a full description of how each resilience factor was derived, including methods and times of measurement.

### Covariates

The analyses were adjusted for relevant demographic, socioeconomic, lifestyle and health variables. These included sex of the child (dichotomous variable); maternal age at birth; birthweight; gestation; maternal smoking in the 2nd trimester of pregnancy (binary yes/no); parity, defined as the number of times that the woman had given birth to a child with a gestational age of 24 weeks or more; ethnicity of the child (coded white/BAME); socioeconomic status based on maternal and partner educational attainment (none/Certificate of Standard Education to University degree); occupational social class as classified by the Office of Population Censuses and Surveys in 1990 (classes I (professional/managerial) to V (unskilled/manual workers)); home ownership at birth (binary rented/owned); marital status at birth (binary married/not married); mother BMI category pre-pregnancy (underweight, normal weight, overweight, obese); and Alcohol Use Disorders Identification Test (AUDIT) measured when the child was 17.5 years (continuous score with higher score indicating higher risk of problem drinking).

### Missing data

Missing data on all ACE items (outcome variables and covariates) were estimated using multiple imputation by chained equations (MI). The proportion of missingness in the analytic sample ranged from 0% to 78.1%. Mothering score and nicotine dependency scores were removed from the analysis due to too high missingness (> 80%). All variables except ID number were used as predictors in the imputation models. MI estimates missing information under the Missing at Random (MAR) assumption [61], yet even under the Not Missing at Random (NMAR) assumption, MI gives less biased results than listwise deletion [96]. If all variables associated with the missing data generation processes are included in the imputation models then missing values can be more plausibly imputed [80]. ACE measures were computed for participants who answered at least 50% of the questions used to derive the binary measures of ACEs [45]. These participants have higher socioeconomic status than the full cohort and including only these participants will lead to lower estimates of ACE occurrence and could result in selection bias [46]. To make the NMAR/MAR assumption more plausible, we included sociodemographic indicators that are associated with missingness; many of the ACEs, NEET status and SDQ outcomes (mother’s home ownership status at birth, mother and partner’s highest educational qualification, maternal age at birth, maternal marital status at birth, birthweight, parity, gestational age, maternal BMI, maternal smoking during pregnancy, alcohol dependency, ethnicity of the child). Given the size of the imputation model and the available computer resources, we imputed twenty datasets using the mice (Multivariate Imputation by Chained Equations) package version 3.11.0 in R -4.0.3 with 20 iterations per dataset [14], and incorporated relevant auxiliary variables. A comparison of observed and imputed data (Table 2) suggests the imputation procedure was conducted consistent to expected missingness patterns. Reflected by a higher missingness rate in more deprived participants [46], disadvantaged sociodemographic indicators (manual social class, low parental education, and rented home tenure) were lower in the original data than the imputed data. (Table 1). ACE exposure estimates are higher in the imputed data, as expected.

**Table 2:** Prevalence estimates and sample characteristics for ACE, SDQ, NEET status and demographic measures in the original ALSPAC data and imputed data

Variable	Original Data (total N = 14,964)			Imputed data (N = 293,880)
	N	% Missing	Mean (sd) for continuous variables, % for categorical variables	Mean (sd) for continuous variables. % for categorical variables
SDQ total difficulties (16years)	5566	62.12	6.1 (4.8)	6.8 (5.3)
Physical abuse	7662	47.86	0.4	3.2
Emotional abuse	7655	47.90	2.1	5.7
Emotional neglect	5728	61.02	15.2	19
Bullying	6841	53.44	14.5	15.5
Violence between parents	7675	47.77	0.8	4
Substance abuse household	3618	75.38	0.1	6.9
Parental mental health Problems	3652	75.15	1.9	10.2
Parent convicted offence	6864	53.29	0.7	2.5
Parental separation	5567	62.11	8.3	13.2
Financial difficulties	7605	48.24	0.9	3.3
Neighbourhood satisfaction	5287	64.02	9.8	12.7
Social support child	6639	54.82	5.2	7.3
Social support parent	6948	52.72	2.3	4.1
Violence between child and partner	3977	72.93	11	14.2
Parent child bond	5378	63.40	5.6	9
Gestation (weeks)	13,788	6.17	39.4 (1.9)	39.4 (1.9)
Social class household (32wk gestation)	8773	40.30		
I—Professional			11.3	9.9
II—Managerial and technical			44.4	40.7
IIIM—Skilled manual			11.7	14.5
IIINM Skilled non-manual			30.2	29.5
IV—Partly skilled			2.2	4.1
V—Unskilled			0.2	1.3
Maternal smoking 2nd trimester	12,984	11.64		
No			80.2	79.5
Yes			19.8	20.5
IQ category	7421	49.50		
Exceptionally low			1.8	3.6
Low			5.3	7.4
Low average			12.1	13.8
Average			43.8	42.2
High average			19.5	16.8
High			9.3	8.5
Exceptionally high			8.1	7.7
Reading speed (reading age)	6823	53.57	10.4 (1.7)	10.2 (1.8)
Reading accuracy (reading age)	6840	53.45	9.9 (1.8)	9.7 (1.8)
Reading comprehension (reading age)	6840	53.45	9.6 (1.7)	9.4 (1.8)
Self esteem: global self-worth	6833	53.50	19.2 (3.4)	19.1 (3.5)
Self Esteem: scholastic competence	6844	53.42	17.0 (3.7)	16.9 (3.7)
Locus of control	7038	52.10		
Externalised			57.3	60
Internalised			42.7	40
Mental flexibility (mean reaction time)	6871	53.24	598.5 (67.6)	602.1 (70.7)
Cognitive skills	8360	43.11	2.0 (0.1)	1.9 (0.1)
Temperament	8393	42.88	17.3 (3.4)	17.2 (3.4)
Grandparent attachment	8118	44.75		
Yes			45.8	46
No			54.2	54
Sibling relationship score	6294	57.17	31.3 (5.5)	31.4 (5.5)
School attendance	8052	45.20		
Over 10 days absent			6.7	8
Less than 10 days absent			93.3	92
School perception	3215	78.12	23.6 (3.9)	23.3 (4.1)
Religion	8077	45.03		
No			44.1	47
Yes			55.9	53
Extra-curricular activities	5571	62.09		
Yes			42.2	38.3
No			57.8	61.7
Friendship	6650	54.74	20.9 (2.2)	20.8 (2.2)
Birthweight (kg)	13,615	7.34	3.4 (0.6)	3.4 (0.6)
Maternal age	13,788	6.17	28.0 (5.0)	28.0 (5.0)
Home ownership status birth	12,406	15.57		
Owned			76	74.3
Rented			24	25.7
Marital status birth	12,915	12.11		
Not married			25.1	25.9
Married			74.9	74.1
Parity	12,757	13.18		
0			44.8	44.3
1			35	34.4
2			14.3	14.3
3			4.2	4.5
4			1.2	1.5
5 +			0.5	0.9
Mother highest education qualification	12,251	16.63		
CSE/None			20.2	21.3
Vocational			9.9	10
O Level			34.6	34.2
A Level			22.5	21.9
Degree			12.9	12.6
Ethnicity	12,163	17.22		
White			97.4	97.1
BAME			2.6	2.9
Maternal BMI category	11,378	22.57		
Underweight			5	5.4
Normal Weight			73.4	72.8
Overweight			16.1	16.1
Obese			5.5	5.7
Partner highest education qualification	11,775	19.87		
CSE/None			26.1	27.9
Vocational			8.5	8.8
O level			22.6	20.9
A level			26	25.2
Degree			18.2	17.3
Sex	14,694	0.00		
Male			51	51
Female			49	49
SMFQ—depressive Symptoms (18yrs)	3305	77.51	6.8 (5.9)	7.1 (6.2)
NEET at 17 years	4052	72.42		
Yes			4.3	9.3
No			95.7	90.7
NEET at 23 years	3997	72.80		
Yes			7	11
No			93	89
AUDIT score at 17years	4108	72.04	7.0 (4.9)	7.2 (5.1)

In the imputed data, emotional neglect was the most common ACE (19.0%), which is in line with previous reported prevalence of emotional neglect as ACE exposure in the UK population [6]. Parent convicted of an offence had the lowest prevalence (2.5%), which is lower than other ACE studies in the UK (4.0 to 4.1%) [6, 48] and the US (7%) [22] but still in line with parental criminal estimates in high-income countries [57]. NEET status at 17 years and 23 years are both higher in the imputed data (but in line with UK estimates for NEET status in the UK in 16–18 year olds in 2007), and slightly lower than reported NEET status for 23 year olds in the UK in 2013 (in the corresponding year to data collection in ALSPAC) [27].

### Statistical analyses

All analyses were performed in R version 4.0.3 (2020–10-10), *RStudio* version 1.3.1093 for Windows. SMFQ and SDQ were both positively skewed (SMFQ skewness = 1.22; SDQ skewness = 1.28) so were square-root transformed to reduce non-normality of the raw data distributions, and meet the assumptions of the predictive linear model (See Additional file 2: Fig. S1). Resilience was derived from the standardised residuals of a multiple linear regression model where binary exposures of ACEs experienced in adolescence (11–16yrs) were regressed on the total difficulties score of the SDQ at 16 years 6 months. The associations between resilience, resilience factors and covariates, and resilience and NEET status and SMFQ outcomes were modelled in separate models. The associations between depressive symptoms at 18, resilience, ACEs and covariates were tested using multiple linear regression. NEET status at 17 and 23 were run as separate binary multiple logistic regression models to test associations with resilience and known demographics previously associated with NEET status. All reported results are from the pooled estimates from the multiple imputed analyses. The standardized regression coefficients reported in the results section resulted from rerunning the final models in the combined dataset containing all the imputed datasets.

## Results

The characteristics of the study sample are summarised in Table 2 and include the original and imputed data. 47.7% of the children were female, with an overwhelming majority of the children being white (97.5%). The majority of the mothers were married (75.8%) and owned their own home at birth (77%). While the prevalence of individual ACEs in the initial sample ranged from 0.1% (substance abuse in the household) to 15.2% (emotional neglect), in the imputed sample the range was from 2.5% (parent convicted of an offence) to 19.0% (emotional neglect).

### Deriving resilience from the residuals of SDQ total difficulties score predicted by ACEs

**Fig. 1:**
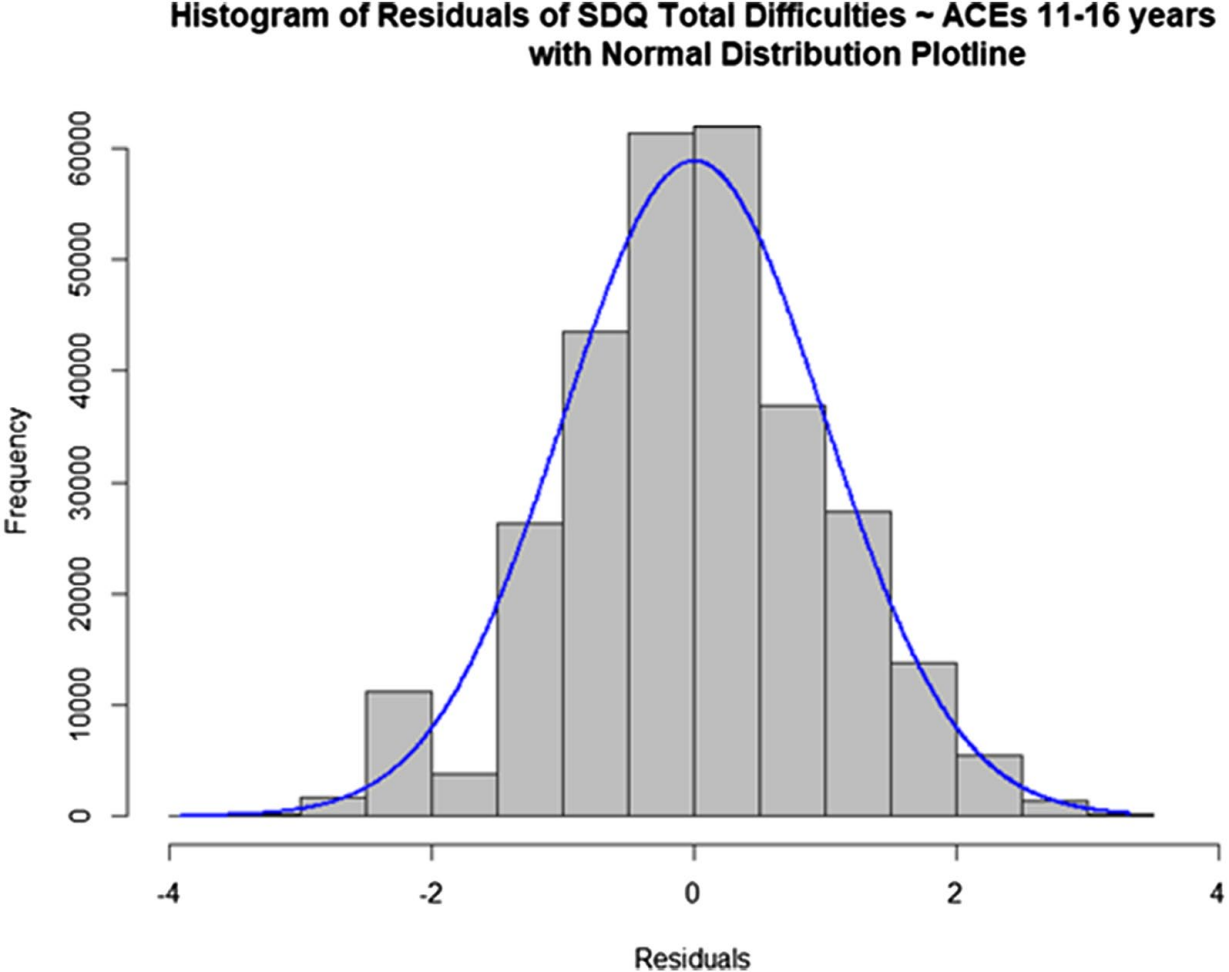
Histogram of the residual variance of the multiple linear regression model: SDQ total difficulties at 16 years ~ ACEs 11–16 years with normal distribution plotline

Eight of the fifteen ACEs were significantly associated with SDQ total behavioural difficulties at 16 years of age. Financial difficulties, emotional abuse, and lack of parent child bond were the strongest predictors of total behavioural difficulties (see Table 3). Resilience was derived using the residuals from the full model (mean = − 0.99, sd = 5.00, range = − 25.34 to 17.63). See Fig. 1 for residual distribution of the multiple linear regression model.

**Table 3:** Adolescent ACEs predicting total behavioural difficulties on strength and difficulties questionnaire

Predictors	SDQ total behavioural difficulties ~ adolescent ACEs					
	Estimates	Std. Beta	Standardized CI	CI	t value	p
Intercept	2.15	0.94	0.93 to 0.94	2.12 to 2.19	121.56	< 0.001
Physical abuse (11–16 years)	− 0.02	− 0.01	− 0.02 to 0.00	− 0.44 to 0.39	− 0.11	0.912
Emotional abuse (11–16 years)	0.30	0.13	0.12 to 0.14	0.06 to 0.55	2.56	0.016
Emotional neglect (11–16 years)	0.17	0.07	0.07 to 0.08	0.08 to 0.26	3.96	< 0.001
Bullying (11–16 years)	0.20	0.09	0.08 to 0.09	0.12 to 0.29	4.78	< 0.001
Violence between parents (11–16 years)	0.05	0.02	0.01 to 0.03	− 0.22 to 0.32	0.37	0.711
Household substance abuse (11–16 years)	0.11	0.05	0.04 to 0.06	− 0.22 to 0.44	0.69	0.497
Parental mental health problems (11–16 years)	0.18	0.08	0.07 to 0.09	− 0.01 to 0.37	1.92	0.066
Parent convicted of offence (11–16 years)	0.03	0.01	− 0.00 to 0.02	− 0.20 to 0.26	0.27	0.789
Parental separation (11–16 years)	0.09	0.04	0.03 to 0.04	− 0.04 to 0.23	1.44	0.162
Financial difficulties (11–16 years)	0.31	0.13	0.12 to 0.14	0.06 to 0.55	2.57	0.016
Neighbourhood dissatisfaction (11–16 years)	0.23	0.10	0.09 to 0.10	0.11 to 0.35	3.85	0.001
No social support—child (11–16 years)	0.27	0.12	0.11 to 0.13	0.12 to 0.42	3.76	0.001
No social support—parent (11–16 years)	0.15	0.07	0.06 to 0.07	− 0.03 to 0.32	1.68	0.103
Violence between child and partner (11–16 years)	0.20	0.09	0.08 to 0.09	0.10 to 0.30	4.00	< 0.001
No parent child bond (11–16 years)	0.31	0.13	0.13 to 0.14	0.17 to 0.46	4.48	< 0.001

Adjusted R^2 =^ 0.091 (0.066—0.119)

### Construct validity—predicting resilience with resilience factors

We assessed the construct validity of the residuals approach by investigating whether previously identified RFs significantly predict resilience as measured by the residuals approach. The multiple linear regression model of resilience predicted by resilience factors and demographic variables is given in Table 4.

**Table 4:** Multivariate linear regression model of resilience predicted by resilience factors and demographic variables

Predictors	Resilience to SDQ total difficulties ~ resilience Factors & demographic variables				
	β	std. Error	CI	t value	p
Intercept	− 21.85	2.49	− 26.94 to − 16.77	− 8.77	< 0.001
Social class IV—partly skilled	0.20	1.14	− 2.15 to 2.54	0.17	0.864
Social class IIINM—skilled non-manual	− 0.17	0.82	− 1.86 to 1.51	− 0.21	0.834
Social class IIIM—skilled manual	0.09	0.88	− 1.71 to 1.89	0.10	0.918
Social class II—managerial and technical	0.10	0.83	− 1.60 to 1.81	0.13	0.901
Social class I—professional	0.40	0.81	− 1.24 to 2.05	0.50	0.620
Maternal smoking (2nd trimester)	− 0.18	0.22	− 0.62 to 0.27	− 0.80	0.431
Birthweight (kg)	0.15	0.15	− 0.15 to 0.45	0.98	0.331
Maternal age (years)	− 0.01	0.02	− 0.04 to 0.02	− 0.71	0.482
Home ownership status birth (owned)	0.15	0.20	− 0.26 to 0.56	0.73	0.469
Marital status birth (married)	− 0.19	0.19	− 0.57 to 0.19	− 1.00	0.322
Parity (1)	0.93	0.15	0.63 to 1.23	6.20	< 0.001
Parity (2)	0.53	0.27	− 0.02 to 1.07	1.97	0.058
Parity (3)	0.92	0.34	0.23 to 1.60	2.70	0.010
Parity (4)	1.59	0.82	− 0.09 to 3.26	1.95	0.062
Parity (5 +)	0.60	1.12	− 1.71 to 2.91	0.54	0.596
Mum highest education—vocational	− 0.23	0.37	− 1.00 to 0.53	− 0.63	0.534
Mum highest education—O level	0.03	0.23	− 0.44 to 0.50	0.14	0.889
Mum highest education—A level	0.18	0.28	− 0.38 to 0.75	0.66	0.512
Mum highest education—degree	0.09	0.30	− 0.51 to 0.69	0.30	0.764
Ethnicity (BAME)	0.57	0.53	− 0.53 to 1.66	1.06	0.297
Maternal BMI (underweight)	− 0.51	0.31	− 1.14 to 0.12	− 1.64	0.109
Maternal BMI (overweight)	0.26	0.25	− 0.24 to 0.76	1.06	0.298
Maternal BMI (obese)	− 0.51	0.41	− 1.36 to 0.33	− 1.25	0.222
Partner highest education—vocational	0.08	0.33	− 0.60 to 0.76	0.24	0.815
Partner highest education—O level	0.02	0.27	− 0.52 to 0.57	0.08	0.937
Partner highest education—A level	− 0.11	0.27	− 0.67 to 0.45	− 0.40	0.689
Partner highest education—degree	− 0.04	0.32	− 0.68 to 0.61	− 0.11	0.912
Gestation (weeks)	− 0.04	0.04	− 0.13 to 0.05	− 0.90	0.373
Sex (Female)	− 0.24	0.16	− 0.56 to 0.07	− 1.56	0.129
Low IQ (70—79)	0.28	0.84	− 1.47 to 2.03	0.33	0.743
Low average IQ (80–89)	0.95	0.74	− 0.58 to 2.49	1.29	0.211
Average IQ (90–109)	1.19	0.79	− 0.44 to 2.82	1.52	0.143
High average IQ (110–119)	1.21	0.71	− 0.25 to 2.67	1.71	0.100
Very high IQ (120–129)	1.47	0.76	− 0.10 to 3.04	1.92	0.066
Exceptionally high IQ (130 +)	1.31	0.76	− 0.25 to 2.87	1.72	0.097
Reading speed (years)	− 0.01	0.05	− 0.12 to 0.10	− 0.16	0.872
Reading accuracy (years)	0.04	0.07	− 0.10 to 0.19	0.62	0.541
Reading comprehension (years)	0.21	0.07	0.06 to 0.35	2.80	0.008
Self esteem: global self-worth	0.08	0.03	0.01 to 0.14	2.25	0.033
Self esteem: scholastic competence	0.03	0.02	− 0.02 to 0.07	1.14	0.261
Locus of control—internalised	0.25	0.16	− 0.07 to 0.57	1.56	0.127
Mental flexibility—middle tertile	0.09	0.17	− 0.24 to 0.43	0.56	0.580
Mental flexibility—fastest tertile	− 0.02	0.17	− 0.36 to 0.33	− 0.09	0.928
Cognitive skills	2.75	0.86	0.97 to 4.53	3.18	0.004
Temperament	0.28	0.02	0.24 to 0.32	13.64	< 0.001
Grandparent attachment—Yes	− 0.04	0.15	− 0.33 to 0.26	− 0.25	0.803
Positive sibling relationship	0.05	0.02	0.02 to 0.09	2.90	0.007
School attendance (< 10 days off)	0.51	0.29	− 0.08 to 1.10	1.75	0.090
School perception	0.13	0.02	0.09 to 0.18	5.64	< 0.001
Religious engagement (Yes)	− 0.06	0.14	− 0.34 to 0.22	− 0.42	0.679
Extracurricular activities—Yes	0.54	0.14	0.26 to 0.82	3.89	< 0.001
Supportive friendships	0.04	0.04	− 0.05 to 0.12	0.96	0.347

Adjusted R^2^ = 0.139 (0.116 – 0.164)

Seven of the 17 resilience factors were associated with higher levels of resilience, and their effect on resilience was in line with predictions based on prior research. Somewhat surprisingly, no socioeconomic factors significantly predicted resilience. Parity is the only demographic variable significantly associated with higher levels of resilience. A parity of 1, i.e. having had just one child, (std. β = 0.15, p < 0.001) and a parity of 3 (std. β = 0.34, p = 0.010) was associated with higher levels of resilience, compared to a parity of 0. Having a parity of 2 trends towards a significant association with higher levels of resilience (p = 0.058).

#### Individual RFs

High cognitive skills at 6 years, 9 months was the strongest predictor of higher levels of resilience (β = 2.75, p = 0.004). Reading comprehension at 9 years was associated with higher levels of resilience at 16 years (β = 0.21, p = 0.008). Having a less emotional temperament at 5 years 9 months was associated with higher levels of resilience at 16 years (β = 0.28, p =  < 0.001). A high degree of global self-worth at 8 years was associated with higher levels of resilience at 16 years (β = 0.08, p = 0.033). Having a very high IQ trended towards a significant positive association (p = 0.066) when compared to an exceptionally low IQ in predicting resilience.

#### Family RFs

The only family resilience factor significantly associated with higher levels of resilience was positive sibling relationship, measured at 11 years, 8 months (β = 0.05, p = 0.007).

#### Community RFs

A positive opinion of school at 11 years and 14 years was associated with higher levels of resilience at 16 years (β = 0.13, p =  < 0.001). Regularly participating in extracurricular activities from 6 to 16 years was associated with higher levels of resilience at 16 years (β = 0.54, p =  < 0.001) when compared with not participating in extracurricular activities.

### Predictive validity

#### Predicting depressive symptoms at 18 years with resilience at 16 years

The predictive validity of resilience when measured using the residuals approach was investigated by comparing the predictive power of the residual measure of resilience with other determinants of psychosocial functioning on depressive symptoms at 18 years as measured by the SMFQ. The multivariate linear regression model of the square root of depressive symptoms at 18yrs, measured by the SMFQ, predicted by resilience, ACEs and demographic variables is given in Table 4.

Resilience significantly predicted a reduction in the risk of having depressive symptoms. A one standard deviation increase in resilience at 16 years was associated with a 0.12 reduced risk of having depressive symptoms at 18 years (std. β = − 0.12, p < 0.001). This is a comparable effect size to the effect of ACEs on depressive symptoms. Experiencing bullying, emotional abuse, parental neighbourhood dissatisfaction or violence between the child and partner in adolescence (11–16 years) was significantly associated with higher levels of depressive symptoms at 18 years (bullying, std. β = 0.15, p < 0.001; emotional abuse, std. β = 0.16, p = 0.012; neighbourhood dissatisfaction, std. β = 0.11, p = 0.018; violence between child and partner, β = 0.15, p = 0.001). In line with previous research on sex differences in adolescent depressive symptoms in ALSPAC [59], and in a Norwegian longitudinal cohort [62], being female is significantly associated with higher levels of depressive symptoms at 18 years (std. β = 0.18, p < 0.001), compared with males. No other demographic indicator was significantly associated with depressive symptoms at 18 years. There were no significant two-way interactions between ACEs and resilience (Table 5).

### Predictive validity

#### Predicting NEET status at 17 and 23 years with resilience at 16 years

We investigated the predictive validity of resilience when measured using the residuals approach by comparing the predictive power of the residuals measure of resilience with other determinants of psychosocial functioning on NEET status at 17 and 23 years. Resilience to SDQ difficulties at 16 years significantly reduces the risk of having NEET status at 17 years and 23 years. The results are summarised in Table 5.

**Table 5:** Multiple linear regression model of depression symptoms, as measured by SMFQ, predicted by resilience, demographic factors and ACEs

Predictors	Depressive symptoms ~ adolescent ACEs, resilience and associated demographics					
	b	Std. β	Std. Error	CI	t	p
Intercept	1.70	0.81	0.52	0.64 to 2.77	3.28	0.003
Resilience	− 0.06	− 0.12	0.01	− 0.07 to − 0.05	− 11.00	< 0.001
Physical abuse (11–16 years)	− 0.43	− 0.17	0.34	− 1.14 to 0.28	− 1.27	0.219
Emotional abuse (11–16 years)	0.39	0.16	0.14	0.09 to 0.69	2.71	0.012
Emotional neglect (11–16 years)	0.09	0.04	0.07	− 0.05 to 0.23	1.34	0.194
Bullying (11–16 years)	0.36	0.15	0.06	0.24 to 0.48	6.27	< 0.001
Violence between parents (11–16 years)	0.29	0.11	0.14	− 0.00 to 0.59	2.02	0.053
Household substance abuse (11–16 years)	0.01	0.01	0.25	− 0.51 to 0.52	0.02	0.983
Parental mental health problems (11–16 years)	0.11	0.04	0.17	− 0.24 to 0.46	0.65	0.523
Parent convicted of offence (11–16 years)	− 0.03	− 0.01	0.26	− 0.57 to 0.51	− 0.12	0.907
Parental separation (11–16 years)	0.10	0.04	0.07	− 0.05 to 0.25	1.36	0.185
Financial difficulties (11–16 years)	− 0.13	-0.04	0.20	− 0.54 – 0.28	− 0.65	0.522
Neighbourhood dissatisfaction (11–16 years)	0.27	0.11	0.11	0.05 to 0.49	2.55	0.018
No social support—child (11–16 years)	0.10	0.04	0.12	− 0.14 to 0.34	0.88	0.387
No social support—parent (11–16 years)	0.15	0.06	0.12	− 0.10 to 0.40	1.21	0.236
Violence between child and partner (11–16 years)	0.38	0.15	0.10	0.17 to 0.60	3.71	0.001
No parent child bond (11–16 years)	− 0.03	− 0.01	0.10	− 0.24 to 0.19	− 0.26	0.800
Social class IV—partly skilled	− 0.16	-0.06	0.27	− 0.72 to 0.41	− 0.58	0.566
Social class IIINM—skilled non-manual	0.03	0.01	0.28	− 0.54 to 0.61	0.11	0.910
Social class IIIM—skilled manual	0.00	0.00	0.28	− 0.57 to 0.58	0.01	0.993
Social class II—managerial and technical	0.00	0.00	0.28	− 0.57 to 0.57	0.01	0.991
Social class I—Professional	0.02	0.01	0.29	− 0.59 to 0.63	0.06	0.95
Maternal smoking (2nd trimester)	0.09	0.04	0.06	− 0.03 to 0.21	1.60	0.121
Birthweight (kg)	0.07	0.02	0.04	− 0.01 to 0.16	1.76	0.086
Maternal age (years)	0.00	0.01	0.00	− 0.00 to 0.01	0.97	0.339
Home ownership status birth (owned)	− 0.05	− 0.02	0.08	− 0.21 to 0.12	− 0.57	0.573
Marital Status Birth (Married)	− 0.08	− 0.03	0.05	− 0.19 to 0.03	− 1.51	0.141
Parity (1)	0.01	0.00	0.04	− 0.08 to 0.10	0.26	0.798
Parity (2)	0.14	0.06	0.07	− 0.01 to 0.29	1.94	0.063
Parity (3)	− 0.08	− 0.03	0.13	− 0.35 to 0.19	− 0.59	0.559
Parity (4)	− 0.22	− 0.09	0.19	− 0.61 to 0.16	− 1.19	0.243
Parity (5 +)	0.12	0.04	0.42	− 0.75 to 0.98	0.28	0.780
Mother highest education—vocational	− 0.15	− 0.06	0.11	− 0.38 to 0.09	− 1.31	0.201
Mother highest education—O level	− 0.01	0.00	0.07	− 0.14 to 0.13	− 0.14	0.890
Mother highest education—A level	− 0.08	− 0.03	0.07	− 0.23 to 0.06	− 1.20	0.238
Mother highest education—degree	− 0.14	− 0.06	0.07	− 0.29 to 0.01	− 1.86	0.069
Ethnicity (BAME)	0.02	0.01	0.14	− 0.27 to 0.31	0.13	0.898
Maternal BMI (Underweight)	0.06	0.02	0.10	− 0.15 to 0.27	0.57	0.574
Maternal BMI (Overweight)	0.01	0.00	0.04	− 0.08 to 0.10	0.18	0.855
Maternal BMI (Obese)	-0.03	-0.01	0.09	− 0.21 to 0.15	-0.34	0.738
Partner highest education—vocational	0.10	0.04	0.08	− 0.06 to 0.26	1.23	0.230
Partner highest education—O level	0.03	0.01	0.07	− 0.11 to 0.17	0.41	0.688
Partner highest education—A level	0.03	0.01	0.05	− 0.07 to 0.14	0.61	0.546
Partner highest education—degree	0.01	0.00	0.08	− 0.15 to 0.17	0.09	0.931
Gestation (weeks)	0.00	0.00	0.01	− 0.03 to 0.02	− 0.22	0.827
Sex (Female)	0.45	0.18	0.04	0.37 to 0.52	12.29	< 0.001
Emotional abuse (11–16 years): resilience	0.00	0.01	0.01	− 0.01 to 0.02	0.43	0.67
Emotional neglect (11–16 years): resilience	0.00	0.00	0.01	− 0.01 to 0.02	0.07	0.946
Bullying (11–16 years): resilience	0.00	0.00	0.01	− 0.01 to 0.01	− 0.08	0.94
Financial difficulties (11–16 years): resilience	0.00	0.00	0.01	− 0.02 to 0.02	0.04	0.966
Neighbourhood dissatisfaction (11–16 years): resilience	0.00	0.00	0.01	− 0.02 to 0.01	− 0.11	0.915
No social support—child (11–16 years): resilience	0.00	0.01	0.01	− 0.01 to 0.02	0.36	0.718
Violence between child and partner (11–16 years): resilience	0.00	− 0.01	0.01	− 0.02 to 0.01	− 0.48	0.629

Adjusted R^2^ = 0.174 (0.146 – 0.203)

**Table 6:** Multiple logistic regression model of NEET status at 17 years predicted by resilience and demographic factors

Predictors	NEET status at 17 years ~ Adolescent ACEs, resilience and associated demographics				
	OR	Std. Error	CI	Statistic	p
Intercept	2.99	4.79	0.11–79.65	0.68	0.500
Resilience	0.93	0.02	0.89–0.97	− 3.68	0.001
Maternal smoking (2nd trimester)	1.00	0.19	0.68–1.48	0.01	0.989
Birthweight (kg)	1.02	0.20	0.68–1.52	0.10	0.925
Maternal age (years)	0.95	0.02	0.91–0.99	− 2.76	0.010
Home ownership status birth (owned)	0.56	0.14	0.33–0.94	− 2.31	0.031
Marital status birth (Married)	0.69	0.17	0.41–1.16	− 1.47	0.154
Parity (1)	1.56	0.30	1.05–2.32	2.33	0.028
Parity (2)	1.69	0.39	1.05–2.73	2.26	0.031
Parity (3)	1.08	0.61	0.33–3.46	0.13	0.898
Parity (4)	2.61	1.68	0.69–9.91	1.48	0.152
Parity (5 +)	3.11	2.30	0.68–14.28	1.54	0.138
Mum highest education—vocational	0.55	0.21	0.25–1.23	− 1.53	0.141
Mum highest education—O level	0.72	0.18	0.43–1.20	− 1.34	0.194
Mum Highest education—A level	0.67	0.21	0.35–1.30	− 1.24	0.226
Mum highest education—degree	0.89	0.29	0.45–1.74	− 0.36	0.722
Ethnicity (BAME)	1.00	0.48	0.37–2.72	0.01	0.992
Maternal BMI (Underweight)	1.60	0.58	0.76–3.38	1.30	0.205
Maternal BMI (Overweight)	1.10	0.23	0.71–1.70	0.45	0.658
Maternal BMI (Obese)	0.98	0.42	0.40–2.39	− 0.04	0.970
Partner highest education—vocational	0.94	0.28	0.52–1.72	− 0.21	0.838
Partner highest education—O level	0.57	0.13	0.36–0.92	− 2.43	0.022
Partner highest education—A level	0.77	0.16	0.50–1.17	− 1.28	0.209
Partner highest education—degree	0.79	0.19	0.49–1.28	− 1.00	0.325
Gestation (weeks)	0.96	0.04	0.87–1.05	− 0.93	0.360
Sex (Female)	0.74	0.12	0.53–1.03	− 1.85	0.075
AUDIT status (17 years)	1.01	0.01	0.98–1.04	0.48	0.632
Emotional abuse (11–16 years)	2.17	1.04	0.80–5.90	1.61	0.123
Emotional neglect (11–16 years)	1.95	0.43	1.24–3.06	3.06	0.005
Bullying (11–16 years)	0.88	0.16	0.61–1.27	− 0.69	0.493
Financial difficulties (11–16 years)	0.65	0.61	0.09–4.65	− 0.46	0.649
Neighbourhood dissatisfaction (11–16 years)	1.22	0.33	0.70–2.12	0.75	0.462
No social support—child (11–16 years)	1.23	0.38	0.65–2.30	0.67	0.507
Violence between child and partner (11–16 years)	1.68	0.42	1.00–2.83	2.05	0.051
No parent child bond (11–16 years)	1.14	0.39	0.56–2.33	0.38	0.705
Emotional abuse (11–16 years): resilience	1.00	0.03	0.94–1.05	− 0.08	0.940
Emotional neglect (11–16 years): resilience	1.00	0.02	0.97–1.03	− 0.06	0.956
Bullying (11–16 years): resilience	1.00	0.02	0.96–1.04	− 0.18	0.857
Financial difficulties (11–16 years): resilience	1.01	0.03	0.94–1.08	0.23	0.821
Neighbourhood dissatisfaction (11–16 years): resilience	1.00	0.02	0.97–1.04	0.26	0.793
No social support—child (11–16 years): resilience	0.99	0.02	0.94–1.04	− 0.50	0.619
Violence between child and partner (11–16 years): resilience	1.00	0.02	0.97–1.04	0.21	0.835
No parent child bond (11–16 yers): resilience	1.00	0.02	0.96–1.04	0.11	0.910

**Table 7:** Multiple logistic regression model of NEET status at 23 years predicted by resilience and demographic factors

Predictors	NEET status at 23yrs ~ Adolescent ACEs, resilience and associated demographics				
	OR	Std. Error	CI	Statistic	p
Intercept	0.48	0.72	0.02–10.34	− 0.49	0.629
Resilience	0.92	0.01	0.89–0.95	− 5.48	< 0.001
Maternal smoking (2nd trimester)	0.84	0.12	0.62–1.13	− 1.18	0.246
Birthweight (kg)	0.91	0.16	0.63–1.32	− 0.50	0.623
Maternal age (years)	0.98	0.02	0.94–1.03	− 0.77	0.447
Home ownership status birth (owned)	0.70	0.12	0.49–0.99	− 2.11	0.043
Marital status birth (Married)	0.77	0.17	0.50–1.21	− 1.18	0.248
Parity (1)	1.18	0.20	0.84–1.66	0.99	0.331
Parity (2)	1.40	0.30	0.90–2.18	1.57	0.128
Parity (3)	1.57	0.56	0.76–3.26	1.28	0.213
Parity (4)	1.07	0.79	0.23–4.92	0.09	0.932
Parity (5 +)	1.19	1.05	0.19–7.40	0.19	0.848
Mum highest education—vocational	0.62	0.15	0.38–1.02	− 1.97	0.059
Mum highest education—O level	0.65	0.12	0.44–0.96	− 2.25	0.032
Mum highest education—A level	0.78	0.20	0.47–1.32	− 0.95	0.349
Mum highest education—degree	0.82	0.24	0.45–1.50	− 0.67	0.508
Ethnicity (BAME)	1.08	0.40	0.51–2.29	0.22	0.830
Maternal BMI (Underweight)	0.77	0.25	0.39–1.49	− 0.82	0.420
Maternal BMI (Overweight)	0.95	0.19	0.63–1.44	− 0.25	0.808
Maternal BMI (Obese)	1.26	0.34	0.72–2.19	0.84	0.409
Partner highest education—vocational	1.01	0.34	0.51–2.01	0.03	0.975
Partner highest education—O level	0.91	0.21	0.57–1.45	− 0.43	0.672
Partner highest education—A level	1.04	0.25	0.64–1.69	0.16	0.877
Partner highest education—degree	1.29	0.33	0.77–2.16	1.02	0.315
Gestation (weeks)	0.98	0.04	0.89–1.07	− 0.53	0.599
Sex (Female)	1.05	0.15	0.78–1.42	0.36	0.718
AUDIT status (17 years)	1.01	0.02	0.97–1.05	0.56	0.578
NEET status (17 years)	2.05	0.65	1.06–3.97	2.26	0.034
Emotional abuse (11–16 years)	1.86	0.82	0.74–4.67	1.39	0.179
Emotional neglect (11–16 years)	1.26	0.26	0.83–1.93	1.13	0.270
Bullying (11–16 years)	1.43	0.22	1.05–1.96	2.34	0.025
Financial difficulties (11–16 years)	2.67	1.30	0.97–7.34	2.01	0.057
Neighbourhood dissatisfaction (11–16 years)	1.34	0.40	0.72–2.49	0.97	0.343
No social support—child (11–16 years)	1.80	0.51	1.00–3.24	2.08	0.049
Violence between child and partner (11–16years)	1.69	0.45	0.97–2.92	1.96	0.062
No parent child bond (11–16years)	1.07	0.35	0.54–2.12	0.22	0.829
Emotional abuse (11–16 years): resilience	1.01	0.03	0.96–1.06	0.38	0.702
Emotional neglect (11–16 years): resilience	0.99	0.02	0.96–1.02	− 0.53	0.598
Bullying (11–16 years): resilience	1.00	0.02	0.97–1.04	0.22	0.823
Financial difficulties (11–16 years): resilience	1.01	0.03	0.95–1.06	0.21	0.834
Neighbourhood dissatisfaction (11–16 years): resilience	1.00	0.02	0.97–1.03	− 0.07	0.941
No social support—child (11–16 years): resilience	1.00	0.02	0.96–1.05	0.07	0.946
Violence between child and partner (11–16 years): resilience	1.00	0.02	0.97–1.03	0.01	0.993
No parent child bond (11–16 years): resilience	1.00	0.02	0.96–1.04	0.11	0.914

The likelihood of having NEET status at 17 years is 7% lower for one SD increase of resilience (OR = 0.92, 95% CI = 0.89–0.97). A mother having one or two previous child(ren) increases the likelihood of her children’s NEET status by 56% (OR = 1.56, CI = 1.05–2.32) or 69% (OR = 1.69, CI = 1.05–2.73) respectively. Being born into a home owned by your parents reduces the likelihood of NEET status at 17 by 44% (OR = 0.56, CI = 0.33–0.94). For every one year increase in maternal age at birth, the likelihood of having NEET status at 17 is reduced by 5% (OR = 0.95, CI = 0.91–0.99). Paternal or partner’s highest educational qualification of O level (OR = 0.57, CI = 0.36–0.92) reduced the likelihood of having NEET status at 17 by 43%, when compared to having no or CSA education. In terms of ACEs, experiencing emotional neglect in adolescence was the only ACE with a significant association, with an increase in the likelihood of NEET status at 17 years (OR = 1.95, CI = 1.24–3.06). Again, there were no significant interactions between ACEs and resilience (Table 6).

The effect of resilience is enduring as the likelihood of having NEET status at 23 years was significantly reduced for each unit increase of resilience (OR = 0.92, 95% CI = 0.89–0.95). Being NEET at 17 is the strongest predictor of NEET at 23, increasing the likelihood just over two times (OR = 2.05, CI = 1.51–4.04). Children born into a home owned by their parents reduces the likelihood of NEET status at 23 by 30% (OR = 0.70, CI = 0.49–0.99). If the mother has O level as the highest educational qualification, the likelihood of having NEET status at 23 was reduced by 35% (OR = 0.65, CI = 0.44–0.96), when compared to having no or CSA education. No other demographic variables in the final model showed a significant effect on NEET status at 23 years.

The experience of bullying during adolescence increased the likelihood of having NEET status at 23 by 43% (OR = 1.43, CI = 1.05–1.96). Having no social support during adolescence increased the likelihood of having NEET status at 23 years by 80% (OR = 1.80, CI = 1.00–3.24). There were no significant interactions between ACEs and resilience (Table 7).

## Discussion

Although numerous articles have used the residuals approach to measuring resilience [2, 10, 17, 20, 25, 49, 72, 86, 97], to our knowledge, no published studies have formally investigated the validity of this approach. Our study has found that the residuals approach to measuring resilience has both construct and predictive validity. Seven resilience factors were associated with higher levels of resilience and their effect on resilience was in line with predictions based on prior research. Resilience significantly predicted a reduction in the risk of having depressive symptoms at 18 years old and predicted a reduction in the likelihood of having NEET status at 17 and 23 years. Surprisingly, no socioeconomic factors were found to be associated with resilience.

### Construct validity

#### Individual resilience factors

We sought to assess the validity of this methodology by investigating whether previously identified resilience factors and demographic factors significantly predict resilience when measured by the residuals approach. To demonstrate the validity of our approach, we investigated whether individual factors previously associated with an increase in resilience significantly predicted resilience as measured by the residual approach at 16 years in our ALSPAC sample. Indeed, we found high cognitive skills (at 6 years 9 months), reading comprehension (at 9 years), high global self-worth (8 years), and a less emotional temperament (5 years 9 months) represent intrinsic individual level RFs that continue to exert their positive effects for some length of time. They could also be described as generative, setting positive cascades in place that develop other contributing factors such as coping styles and emotion regulation [63]. A less emotional temperament in childhood, described as biologically-based individual differences in reactivity and regulation [83], was a significant predictor of resilience at 16 years. These findings are consistent with previous research that has found children with less emotional temperaments are less reactive to stressors, better able to regulate their feelings of sadness and anger, more likely to maintain positive adaptation and activate flexible coping strategies to deal with adversity [18, 67, 73]. The finding that temperament in childhood predicts resilience in adolescence therefore supports the construct validity of the residual measurement of resilience.

High cognitive skills have previously been associated with positive adaptation in the face of adversity [34, 51], predictive of lower levels of psychiatric disorders, lower rates of conduct problems and higher levels of overall functioning [65]. While having a high IQ was just below the significance threshold in our model predicting resilience, high cognitive skills and high reading comprehension significantly predicted resilience. Having well-developed verbal cognitive abilities could allow children to use verbal strategies to mediate conflict, leading to more circumstance-appropriate behavioural choices and a larger range of coping strategies [13]. In addition to cognitive skills, high global self-worth at 8 years was associated with higher levels of resilience at 16 years. Self-worth is an intrapersonal characteristic that has been previously reported to impact an individual’s potential for resilience [24, 82]. Individuals with high self-worth have high amounts of self-respect, and have positive feelings about themselves, their environment and their ability to deal with life’s challenges, focussing on their strengths [84, 98]. Higher self-worth has been linked to positive cognitive reappraisal [91], an underlying mechanism that protects against stressors and mediates RFs via cognitive processes [54].

### Family resilience factors

In terms of family resilience factors, previous studies have had broad support for high quality caregiver relationships and stable family environments [1, 34, 40]. We found an association between positive sibling relationship and resilience. High-quality sibling relationships are a unique context which can have a direct impact on one another’s socioemotional development, behaviour and adjustment, relevant to resilience [29, 69]. It is interesting to note that women who have had one or three previous children, and two just below the threshold, was also significantly associated with an increase in resilience in their offspring, which may suggest that having siblings increases resilience in adolescence. This enduring association between multiparous (giving birth previously) and resilience could also be related to the extensive physiological, hormonal, and emotional changes experienced by the mother during pregnancy and the postpartum period. The hormonal fluctuations required for the maintenance of pregnancy are unmatched by any other neuroendocrine events in a healthy female’s lifetime [12], with dramatic changes also evident in metabolic, immune, cardiovascular, pulmonary, haematological and neurobiological systems [26]. Despite parity being commonly included as a covariate in studies of psychological outcomes of children of multiparous women, the origins of parity-associated differences in these outcomes remain poorly defined. It is possible that the observed differences reflect effects of prior pregnancy on adaptation to subsequent pregnancies/children. However, well-documented increased activation of the Hypothalamic–Pituitary–Adrenal (HPA) axis (e.g. [19].) and sympathetic nervous system [28] in pregnancy is consistent with well-described physiological responses to psychological stress, supporting the role of psychological factors [35]. Maternal mental health problems are also less common following a multiparous pregnancy (e.g. [50]). Specifically related to resilience, parity has been associated with a modification in disease risk and progression of multiple sclerosis, depression, stroke and Alzheimer’s disease in the mother [26]. Disentangling the mechanisms by which maternal parity is associated with adolescent resilience warrants further investigations.

We found little evidence in support of the relationship between grandparent attachment and resilience, perhaps due to the maternal self-reports used in ALSPAC. Although there is some evidence of detection bias, there is evidence of intergenerational transmission of child abuse [101]. An abusive parent may have been subjected to abuse by their own parent, hence the limited evidence for grandparents exerting a positive influence and predicting resilience. Our construct of the maternal parenting score within ALSPAC had such high missingness (> 80%) that it had to be removed from the missing data analysis. Future research would benefit from including some measure of positive parental engagement as a protective factor.

### Community resilience factors

Within our framework of community level resilience factors, factors relating to school, including positive opinion of school and regular participation in extracurricular activities were associated with higher levels of resilience. These extrinsic school-based factors are in keeping with the dynamic model of resilience, which conceptualizes resilience not as an individual trait but a process resulting from interactions across the life span, dependent upon context and resources [16, 53, 85]. Ungar [94] proposes that when stressors are particularly high, environmental factors become more critical for a person’s resilience than individual characteristics or cognitions.

### Lack of association between socioeconomic factors and resilience

None of the socioeconomic factors were significantly associated with resilience. This is a somewhat surprising result given that health is well established to be socioeconomically stratified, with those in socioeconomically disadvantaged groups at a greater risk of negative physical and psychological outcomes when compared with socioeconomically advantaged groups [66]. Adult socioeconomic advantage has been previously associated with adult resilience, measured at 60–64 years using the residuals methodology [20], yet there is scant evidence of childhood socioeconomic advantage being associated with adolescent resilience. If socioeconomic disadvantage is a major determinant of health, then emotional and cognitive responses to this inequality are of crucial importance and it is likely that the residuals approach is capturing these responses, rather than capturing socioeconomic dis/advantage itself. Future research using the same methodological techniques for measuring resilience could assess whether child socioeconomic disadvantage followed by adult socioeconomic advantage (i.e., upward intergenerational social mobility) is associated with greater resilience. This would support the theory of ‘steeling’ i.e., developing resilience through exposure to mild aversity in early life, which is supported by positive results in animal models (e.g. [64]) yet has limited results in humans [85].

### Implications for interventions

Our study provides support for construct validity of the residuals approach to measuring resilience and suggests some key areas that have important implications for policy, practice, and future work. We note that while the results of this study may be informative for population-level or structural policies, we are not individualising the problem or suggesting to place the onus on individuals to act. All of our recommendations for targeted levels of intervention are within the structural social context in which the children are exposed to ACEs (see [55], for further discussion). While many of the individual RFs we measured are not easily modifiable, there is scope for intervention researchers to have success in enhancing language development [36], cognitive and social-emotional development [89] and global self-worth through physical activity [41]. Intervening in sibling interactions may be useful to encourage high-quality sibling relationships, with two prevention programs already in place in the US (*More Fun with Sisters and Brothers* [56] and *Siblings are Special* [30]). At the community level, our study suggests the school environment is the most important area for policy to focus on and, given that the key protective factors in our study were identified between 5 and 14 years, individuals may particularly benefit from interventions in primary school, particularly school-based strategies that offer a range of extracurricular activities and enable children to feel more positive about school. Policy could target areas that encapsulate multiple protective factors and their intertwined relationships together. For example, the link between physical activity, global self-worth and adaptive cognitive reappraisal [41, 78] could benefit from extracurricular sports programs.

### Predictive validity—depressive symptoms

We investigated the predictive validity of resilience by comparing the predictive power of the residuals method of quantifying resilience with other determinants of psychosocial functioning on two outcomes. Adjusting for ACEs and other sociodemographic factors associated with increased depressive symptoms, resilience was significantly associated with reduced depressive symptoms at age 18, the first outcome in the predictive validity analyses (std. β = -0.12, p < 0.001). These results strongly support the predictive validity of the residuals method of measuring resilience. The identification of resilience as a specific protective factor associated with lower reports of depressive symptoms can inform the development of prevention and treatment interventions for depression. Specifically, the strategies mentioned above promoting resilience in all children, not just those exposed to ACEs, would be beneficial. Additionally, measuring an adolescent’s resilience to SDQ at 16 may be highly informative. Individual differences in resilience scores may have consequences for tailoring prevention interventions for psychiatric disorders. Further research is needed to explore to what extent this measure of resilience has predictive value for prevention and/or clinical intervention.

### Predictive validity—NEET status

In the second predictive validity analysis, resilience also predicted a reduced likelihood of NEET status at both 17 years (OR = 0.93, 95% CI = 0.89–0.97) and 23 years (OR = 0.92, 95% CI = 0.89–0.95). While these are modest effect sizes, they are in line with effect sizes previously associated with the predictive validity of self-assessed resilience [15] and the social competence resilience factor of the resilience scale for adolescents [44]. The continued stable effect of resilience on reduced likelihood of NEET status from 17 to 23 shows that the predictive validity of resilience is enduring. These results indicate that resilience has value as a predictor of both depressive symptoms and risk of NEET status.

### Research and clinical implications

There are research and clinical implications that can be derived from this study. First, the residuals approach to measuring resilience has both construct and predictive validity: it is a measure of the current resilience of an individual, where resilient functioning refers to better mental wellbeing compared to other individuals with similar ACE exposures. Accordingly, resilience researchers can benefit from this measurement of resilience to determine specific resilience factors at the individual, family and community level that are associated with higher levels of resilience. In addition, this measure of resilience can be used as an independent variable to predict various outcome variables such as psychosocial outcomes and overall functioning.

The greatest strength of this measurement of resilience is its ability to be derived from a simpler computational framework that does not require specialised latent variable modelling software, which therefore supports the widespread application of this method. Because this measure is data-driven, it is a measure of the current resilience of an individual, where resilient functioning refers to better mental wellbeing compared to other individuals with similar ACE exposures. The derived measure is influenced by the specific variables used and provides an individual operationalisation of resilience that is relatively simple to compute.

The novelty of this method of quantifying resilience is not that it demonstrates the protective effects of resilience factors. Extensive previous research on resilience factors have shown the positive influence of resilience on important outcomes [52, 58]. Instead, its originality and significance lie in its ability to advance two key research areas that cannot be adequately studied using other measures: (1) mechanisms of resilience and (2) efficacy of interventions designed to increase resilience.

First, the mechanisms underlying the protective effects of resilience are best examined with a quantitative, individual-specific variable that represents the sum of the resilience construct. Proxy measures may represent one small aspect of an individual’s total resilience, which includes a vast array of life experiences or adversity exposure but are difficult to measure. Extracting a quantitative measure of resilience that is not rooted in any one definition or measured by one static tool, is a step towards identifying underlying general resilience mechanisms [49, 54].

Second, this quantitative measure of resilience can be measured longitudinally and used as an ongoing measure of change through therapeutic processes. The ability to assess an individual’s resilience at the outset of intervention provides a beneficial starting point for strength-based, individual focused care. Extracting a measure of resilience that is sensitive to change can better inform these potential interventions. Similarly, by quantifying resilience at multiple time points, one can characterise individual differences in the variation of resilience and ascertain the impact of resilience factors at the individual, family and community level at varying timepoints across the life course. Future studies are needed to explore this.

## Limitations

There are some limitations in the present study that must be acknowledged. First, as with most longitudinal cohorts, there was attrition in all outcomes. Whilst we attempted to minimize the impact of this using multiple imputation with chained equations, this approach cannot remove bias completely. Secondly, our dataset of SDQ outcomes was derived from maternal reports but the parents may underestimate psychosocial problems in adolescence [95]. However, mean ALSPAC scores are similar to national levels [71]. Thirdly, the results found here may be unique to the ALSPAC cohort, a cohort that is very white, with a higher proportion of married mothers who own their own home than the rest of the general population. We therefore need to expand and diversify the sample to allow for these results to be translatable at the population level. To increase the reliability of this measure of resilience, we propose a replication study in a different dataset. Finally, the correlational design cannot determine causal relations, and prospective or experimental studies are needed.

## Conclusions

In sum, this study has shown the construct and predictive validity of quantifying resilience as residual variance in a psychosocial outcome. It has the potential to advance research into the mechanisms and modifiability of resilience. A key next step in applying this method is to learn how a residual resilience variable interacts with stressors over time.

## Supplementary Information

**Additional file 1: Table S1**. Resilience Factors in ALSPAC

**Additional file 1: Fig. S1**. Histograms comparing pre and post transformation distributions of SDQ total difficulties at 16yrs.

## Data Availability

The ALSPAC dataset is available to all researchers on application to ALSPAC data and samples. Please note that the study website contains details of all the data that is available through a fully searchable data dictionary and variable search tool: http://www.bristol.ac.uk/alspac/researchers/our-data/.

## Abbreviations

ACE: Adverse childhood experiences
ALSPAC: Avon longitudinal study of parents and children
AUDIT: Alcohol Use Disorders Identification Test
BAME: Black, Asian and Minority Ethnic
CSA: Compulsory School Age
MAR: Missing at Random
NMAR: Not Missing at Random
MI: Multiple Imputation
NEET: Not in Employment, Education or Training
RF: Resilience Factors
SDQ: Strength and Difficulties Questionnaire
SMFQ: Short Moods and Feelings Questionnaire
