# Supplementary material for: The validity of the residuals approach to measuring resilience to adverse childhood experiences
Source: Child Adolesc Psychiatry Ment Health. 2022 Mar 1;16:18. doi: 10.1186/s13034-022-00449-y (PMC8889660; doi:10.1186/s13034-022-00449-y)
Supplement: Supplementary file 1 — Additional file 1: Table S1. Resilience Factors in ALSPAC [file 13034_2022_449_MOESM1_ESM.docx]

*Supplementary Table 1: Resilience Factors in ALSPAC*

|  | Measure | Method of measurement | Type of Variable | Setting | Hypothesised resilience factor |
| --- | --- | --- | --- | --- | --- |
| *Individual* | **Gender** | Gender | Binary  (female/male) | n/a | Female gender |
|  | **Mental Flexibility** | Stop Signal Task of response inhibition at age 10 years. Mean response times and stop signal response times used as measure. Reverse coded so higher scores reflect better inhibition ability | Continuous | Research clinic | High mental flexibility |
|  | **Reading Skills and Accuracy** | Test of reading skills, accuracy and comprehension administered to the child at 9years, conducted by trained psychologists and speech therapists using a revised Neale Analysis of Reading Ability (NARA II) | Continuous | Research clinic | High linguistic functioning |
|  | **Cognitive function** | Short form of the Wechsler Intelligence Scale for Children III (Wechsler, 1992), an IQ test administered to the child at 8 years 6 months. | Continuous | Research clinic | High IQ |
|  | **Cognitive skills** | Summary score derived from 15 questions answered by the mother on cognitive skills the child is capable of at age 81 months, such as counting, reading, recognizing words, interest in books. | Continuous | Questionnaire | High cognitive skills |
|  | **Locus of control** | 12 item shortened version of the Nowicki-Strickland Internal-External scale for preschool and primary children (Nowicki & Duke, 1974), administered to the child at 8 years 6 months. A summary score with two levels was created by dividing by the mean. | Binary (internal/external) | Research clinic | Internal locus of control |
|  | **Temperament** | 5 item Emotionality subscale of the Emotionality Activity Sociability scale (Buss & Plomin, 1984), reported by the mother when the child was 5 years 9 months. Reverse coded | Continuous | Questionnaire | Less emotional temperament |
|  | **Self Esteem** | 12-item shortened form of Harter’s Self Perception Profile for Children (Harter, 1985) comprising the global self-worth and scholastic competence subscales administered when child was 8 years | Continuous | Research clinic | High self esteem |
| *Family* | **Grandparent relationship** | Mother asked whether child was particularly attached to grandparent when the child was 7 years 7 months. And 10 years | Binary  (yes/no) | Questionnaire | Close attachment to grandparent |
|  | **Maternal parenting** | Summary score derived from 18 questions answered by the mother on the frequency she interacts with the child at ages 6mths, 18mths, 24mths, 38mths, 81mths, 9 years, and 11 years. Interactions included bathing, making things, singing to, reading to/with, playing with toys with, cuddling, actively playing, taking to park, putting to bed, doing other activity, drawing or painting with child, preparing food with child, shopping, classes, sports, homework, talking to. | Continuous | Questionnaire | High maternal care |
|  | **Sibling interaction** | Summary score derived from 7 questions answered by the mother on the frequency the child does different activities with their siblings when the child was 11 years 8 months. Activities included playing, reading, drawing, going out, talking, eating and playing sport. | Continuous | Questionnaire | Positive sibling relationship |
| *Community* | **School attendance** | Total number of days off school the child had taken in the last year for both health & non-health reasons, answered by the mother when the child was 7 years 7 months. A summary score with two levels was created by dividing by the 90^th^ quartile (10 days off). | Binary  (≤ 10/>10 days off) | Questionnaire | High school attendance with no more than 10 days off in the last year |
|  | **Perception of school** | Summary score derived from questions answered by the child aged 11yrs 2 months and 14 years 1 month on how strongly they agreed with seven positively stated opinions of school using a five-point Likert scale. Common stem was “my school is a place where I…” with consequent statements: …really like to go each day, …like to be, …feel proud to be a pupil, …have a lot of fun, …enjoy what I do, …get excited about the work I do. | Continuous | Questionnaire | Positive opinion of school |
|  | **Religion** | Summary measure derived from two questions answered by the mother on whether the child engaged with religion by praying or attending a place of worship at 9 years 7 months. | Binary  (yes/no) | Questionnaire | Engagement with religion |
|  | **Friendship** | Summary score derived from five questions based on a shortened version of the Friendships questionnaire from the Cambridge Hormones and Moods Project (Goodyer, Wright, & Altham, 1990), answered by the child age 12 years 6 months. Questions related to the child’s overall satisfaction with friends and the number of friends, frequency the child sees friends outside school, whether the child can talk to friends about problems & whether friends are understanding. | Continuous | Research clinic | Supportive friendships |
|  | **Extracurricular activity** | Summary measure derived from two questions answered by the mother on whether the child  regularly attended classes/clubs for extracurricular activities like sports, dance etc. or attended special groups like beavers, scouts when the child was 6 years 9 months, 9 and 16 yrs | Binary  (yes/no) | Questionnaire | Regular participation in extracurricular activities |
|  |  |  |  |  |  |
