# Supplementary figures and images for: The validity of the residuals approach to measuring resilience to adverse childhood experiences
Source: Child Adolesc Psychiatry Ment Health. 2022 Mar 1;16:18. doi: 10.1186/s13034-022-00449-y (PMC8889660; doi:10.1186/s13034-022-00449-y)

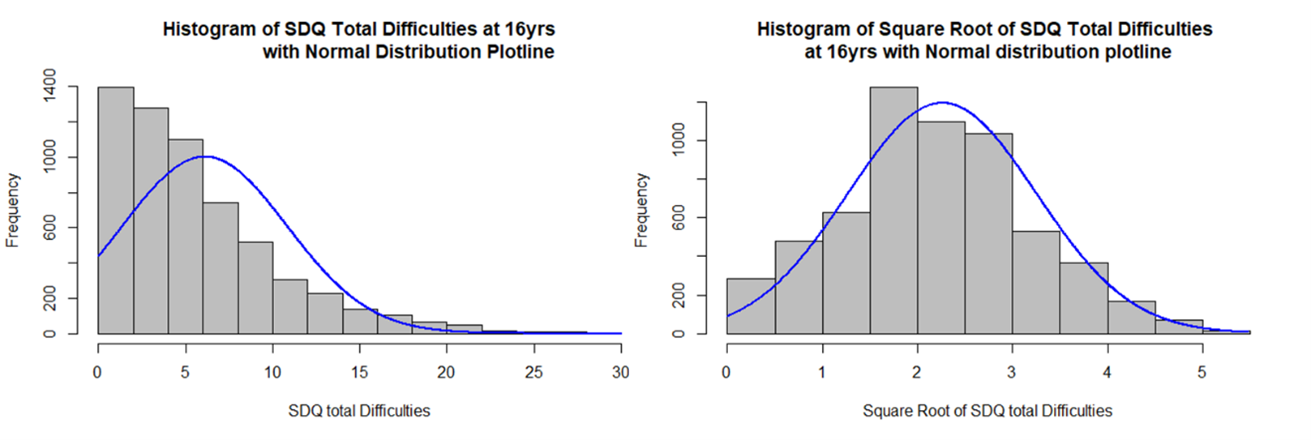

Supplement: Supplementary file 2 — Additional file 1: Fig. S1. Histograms comparing pre and post transformation distributions of SDQ total difficulties at 16yrs. [file 13034_2022_449_MOESM2_ESM.tif]
